# Change in Biomarker Profile After Neoadjuvant Chemotherapy is Prognostic and Common Among Patients with HER2+ Breast Cancer

**DOI:** 10.1245/s10434-024-15889-3

**Published:** 2024-08-08

**Authors:** Julia Tchou, Soumy Gottipati, Macy Goldbach, Molly Baxter, Sara Venters, Ron Balassanian, Poonam Vohra, Diego Gonzalves, Zahra Ahmad, Anupma Nayak, Judy C. Boughey, Rita A. Mukhtar, Yunn-Yi Chen

**Affiliations:** 1 Division of Breast Surgery, Department of Surgery, Perelman School of Medicine, University of Pennsylvania, Philadelphia, PA USA; 2 Abramson Cancer Center, Perelman School of Medicine, University of Pennsylvania, Philadelphia, PA USA; 3 Department of Surgery, University of California San Francisco, San Francisco, CA USA; 4 Department of Laboratory Medicine, University of California San Francisco, San Francisco, CA USA; 5 Department of Pathology, University of California San Francisco, San Francisco, CA USA; 6 Department of Pathology, Perelman School of Medicine, University of Pennsylvania, Philadelphia, PA USA; 7 Department of Surgery, Mayo Clinic, Rochester, MN USA

## Abstract

**Background:**

Rates of pathologic complete response (pCR) after neoadjuvant chemotherapy (NAC) for breast cancer have improved, especially among human epidermal growth factor 2-positive (HER2+) and triple-negative subtypes. The frequency and significance of biomarker profile change in residual disease are unclear. This study aimed to determine the rate of biomarker profile changes after NAC and the impact on clinical outcomes in a contemporary cohort.

**Methods:**

Upon institutional review board approval, the study identified 634 consecutive patients treated with NAC between 2010 and 2022 at two academic institutions. The study cohort was focused on patients with residual disease who underwent biomarker profile retesting. Biomarker profile change for each subtype was compared across groups using Fisher-Irwin tests. Cox Proportional Hazards Model and Kaplan-Meier plots were performed to evaluate the association of changed versus unchanged biomarker profile with event-free survival.

**Results:**

Biomarker retesting was performed for 259 (61.4 %) of 422 patients with residual disease. Biomarker profile change occurred in 18.1 % overall and was significantly higher among those with pre-NAC HER2+ disease (32.7 %, 17/52) than among those with HER2–disease (14.5 %, 30/207) (*p* = 0.004). Conversion of pre-NAC biomarker profiles of HR+HER2– and HR+HER2+ to triple-negative breast cancer (TNBC) post-NAC may be associated with worse event-free survival, hazard ratios of 2.23 (95 % confidence interval [CI], 0.90–5.53; *p* = 0.08), trending toward significance, and 36.7 (95 % CI, 2.2–610.8; *p* = 0.01), respectively.

**Conclusions:**

The results from one of the largest contemporary cohorts demonstrated that biomarker profile change in patients with residual disease after NAC was common. Furthermore, specific biomarker profile change in residual disease may have prognostic value. These findings strengthen the rationale for routine re-testing of biomarkers in residual disease after NAC.

**Supplementary Information:**

The online version contains supplementary material available at 10.1245/s10434-024-15889-3.

The expression status of estrogen receptor (ER) or progesterone receptor (PR) in at least 1 % in breast tumor cells is collectively termed hormone receptor-positive (HR+). The expression of HR in combination with human epidermal growth factor 2 (HER2) is clinically used to classify breast cancer into four biomarker profiles or subtypes as follows: HR+HER2–, HR+HER2+, HR–HER2+, HR–HER2– (also known as triple-negative breast cancer or TNBC). In clinical practice, these four biomarker profiles/subtypes have been synonymous with the molecular breast cancer subtypes defined by distinct transcriptomic profiles.^1^ Treatment recommendations are heavily dependent on the expression status of HR and HER2 biomarkers in the initial breast cancer diagnosis. The biomarker profile has become the cornerstone in determining systemic therapy and treatment sequence, specifically upfront surgery versus neoadjuvant chemotherapy (NAC).

Within the last decade, the introduction of dual HER2-targeted therapy in 2013^2–5^ and immune checkpoint blockade therapy in 2020^6^ combined with NAC has revolutionized the treatment of patients with early-stage (≥cStage 2) HER2+ and TNBC disease, respectively. These advances in neoadjuvant therapy have resulted in improved pathologic complete response (pCR) and clinical outcomes. The frequency and significance of biomarker profile change in residual disease is unclear.

The primary objective of this study was to determine the proportion of patients with biomarker profile change in residual disease after NAC in a contemporary era. The secondary objective was to determine whether biomarker profile change in residual disease may have an impact on event-free survival.

## Methods

Upon IRB approval, we identified 634 consecutive patients with non-metastatic breast cancer treated with NAC between 2009 and 2022 at two large academic institutions. For the cohort from the East Coast site, we identified all consecutively treated breast cancer patients who received NAC between 2016 and 2020 from our prospective breast cancer database and met the specified criteria. For the cohort from the West Coast site, we identified all consecutively treated breast cancer patients who participated in the I-SPY2 clinical trial that enrolled patients with high-risk early-stage breast cancer (>2.5 cm in size), who were randomized to standard of care neoadjuvant chemotherapy or novel agents in an adaptive randomized platform.

**Fig. 1:**
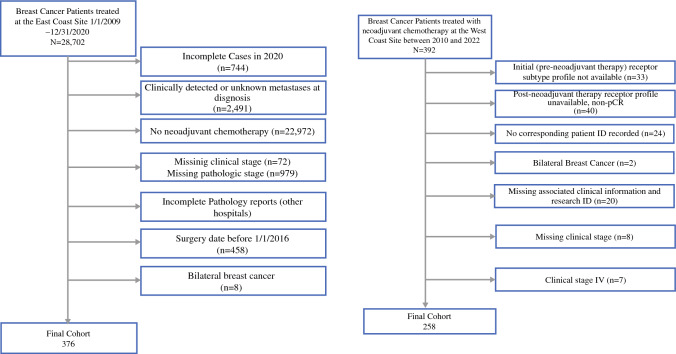
East coast and west coast sites consort diagram

The inclusion and exclusion criteria are summarized in Fig. 1. The clinical characteristics, including event-free and overall survival outcomes, were collected by chart review. Pre-NAC biomarker profiles were grouped according to HR and HER2 status as follows: HR+HER2+, HR+HER2–, HR–HER2+, and HR–HER2–. A biomarker profile was considered changed if either HR or HER2 changed from positive to negative or from negative to positive as defined by American Society of Clinical Oncology (ASCO)/College of American Pathologists (CAP) guideline.

### Statistical Analysis

Clinical characteristics were compared between groups (non-pCR and pCR as well as changed vs unchanged) using Fisher-Irwin and *t* tests. The Cox Proportional Hazards Model was used to evaluate the association of changed versus unchanged post-NAC biomarker profile in residual disease with event-free survival for each of the four pre-NAC biomarker profiles. Kaplan-Meier plots were constructed to compare disease-specific outcomes in each of the four pre-NAC subtypes according to changed versus unchanged biomarker profile. Statistical analyses were performed using STATA version 16/SE (StataCorp LLC, College Station, TX, USA).

## Results

The clinical characteristics of the overall cohort (*n* = 634) are summarized in Table 1. Of the 634 patients, 212 (33.4 %) had pathologic complete response (pCR). The majority of the patients (94 %, 597/634) had clinical stage II disease or higher. The patients with HR+HER2– (*n* = 250) or TNBC ((*n* = 163) disease comprised 39.4 % and 25.7 % of the overall cohort, respectively. The pCR rate differed significantly among the four breast cancer subtypes as follows: HR+HER2- (14.4 %), HR+HER2+ (43.4 %), HR-HER2+ (71.6 %), and TNBC (39.3 %) (*p* < 0.01). As expected, the patients with pCR had better clinical outcomes, with an unadjusted death rate of 6.6 % compared with 16.6 % for those without pCR (*p* < 0.01).

**Table 1:** Clinical characteristic of overall patient cohort treated with neoadjuvant chemotherapy

	Total		no pCR		pCR		p-value
	634		423		211		
Age at diagnosis in years, median (IQR)	49.0 (19.0)		49.0 (19.0)		46.0 (19.0)		**< 0.01**
*Race*							
White	464	73.2%	317	74.9%	147	69.7%	0.33
Black	88	13.9%	58	13.7%	30	14.2%	
Asian/PI	58	9.1%	35	8.3%	23	10.9%	
Other/NA	24	3.8%	13	3.1%	11	5.2%	
*Clinical T stage*							
T0	3	0.5%	0	0.0%	3	1.4%	
T1	62	9.8%	42	9.9%	20	9.5%	**< 0.01**
T2	354	55.8%	216	51.1%	138	65.4%	
T3	172	27.1%	137	32.4%	35	16.6%	
T4	43	6.8%	28	6.6%	15	7.1%	
*Clinical N stage*							
N0	265	41.8%	165	39.0%	100	47.4%	**< 0.01**
N1	301	47.5%	213	50.4%	88	41.7%	
N2	29	4.6%	17	4.0%	12	5.7%	
N3	39	6.2%	28	6.6%	11	5.2%	
*Breast cancer subtype*							
HR+ HER2-	250	39.4%	215	50.8%	35	16.6%	**< 0.01**
HR+ HER2+	122	19.2%	69	16.3%	53	25.1%	
HR- HER2+	67	10.6%	19	4.5%	48	22.7%	
TNBC	163	25.7%	99	23.4%	64	30.3%	
Unknown	32	5.0%	21	5.0%	11	5.2%	
*Histological subtype*							
IDC	518	81.7%	333	78.9%	185	87.3%	**0.02**
ILC	36	5.7%	32	7.6%	4	1.9%	
Mixed	38	6.0%	28	6.6%	10	4.7%	
Other	24	3.8%	18	4.3%	6	2.8%	
Mammary	11	1.7%	8	1.9%	3	1.4%	
Unknown	7	1.1%	4	0.9%	3	1.4%	
*Type of surgery*							
Lumpectomy	277	43.7%	176	41.7%	101	47.6%	0.30
Mastectomy	355	56.0%	246	58.3%	109	51.4%	
Unknown	2	0.3%	1	0.2%	1	0.5%	
Follow up^a^ (months), median (IQR)	51.1 (38.0)		48.6 (37.6)		54.4 (36.5)		0.05
Total recurrence	117	18.5%	99	23.4%	18	8.5%	**< 0.01**
*Recurrence type*							
Local/regional	31	4.9%	28	6.6%	3	1.4%	**0.39**
Distant metastasis	86	13.6%	71	16.8%	15	7.1%	
Total deceased	84	13.2%	70	16.5%	14	6.6%	**< 0.01**

Bold values indicate significant difference (*p* < 0.05)

The biomarker profile of residual tumor was retested for 61.4 % (259/422) of the patients without pCR. The biomarker retesting rate differed between the West Coast and East Coast sites. At the West Coast site, retesting was performed for 352 (90 %) of the 392 patients with residual disease. Insufficient tumor was the most common reason why further biomarker profile testing was not performed at this site. Of the 169 patients with residual disease after NAC, 23 (13.6 %) had biomarker profile change.

At the East Coast Site, retesting was performed for only 90 (37.3 %) of the 241 patients with residual disease. To understand further why biomarker profile re-testing was not performed more consistently, we performed chart review focused on seven surgeons (4 surgeons at the main center and 3 surgeons from an affiliated center). Biomarker profile retesting was performed for 50 (60 %) of the 83 patients at the main site, whereas only 21 (24 %) of the 86 patients at affiliated sites underwent retesting. These results reflected the inconsistent practice pattern in biomarker profile retesting within a single academic center and highlighted the need to implement the policy for biomarker profile retesting uniformly across all sites.

The 259 patients in our final study cohort were further stratified into those with and those without clinically significant biomarker profile change, defined as having one or both HR and HER2 changed from positive to negative or vice versa. Biomarker profile change was noted in 18.1 % (47/259) of the patients. The clinical characteristics of those with and those without biomarker profile change are summarized in Table 2. The two subgroups had similar clinical characteristics except that the proportion of tumors with biomarker profile change may have been of higher cT and cN stages and may have been significantly higher among those with pre-NAC HER2+ disease (32.7 %,17/52) than among those with pre-NAC HER2– (14.5 %,30/207) disease (*p* < 0.01). The number of patients with changed biomarker profile post-NAC from each of the four pre-NAC biomarker profiles is summarized in a 4 × 4 array (Table 3). Notably, the proportion of pre-NAC HR+HER2+ and HR–HER2+ patients with a biomarker profile change was 31.0 % (13/42) and 40.0 % (4/10), respectively, whereas the proportion of pre-NAC HR+HER2– and TNBC patients with a post-NAC biomarker profile change was 17.9 % (25/140) and 7.5 % (5/ 67), respectively (*p* = 0.003).

**Table 2:** Clinical characteristics of patients with residual disease stratified by biomarker profile unchanged vs. changed

	Total		Biomarker profile unchanged		Biomarker profile changed		p-values
	259		212		47		
Age at Diagnosis, median (IQR), years	48.0 (19.0)		48.0 (19.3)		50.0 (17.0)		0.52
Race							
White	193	74.5%	166	78.3%	32	68.1%	0.15
Black	29	11.2%	20	9.4%	9	19.1%	
Asian/PI	26	10.0%	23	10.8%	3	6.4%	
Other/NA	11	4.2%	8	3.8%	3	6.4%	
*Clinical T stage*							
T1	18	6.9%	13	6.1%	5	10.6%	**< 0.01**
T2	146	56.4%	120	56.6%	26	55.3%	
T3	85	32.8%	72	34.0%	13	27.7%	
T4	10	3.9%	7	3.3%	3	6.4%	
*Clinical N stage*							
N0	103	39.8%	86	40.6%	17	36.2%	**< 0.01**
N1	128	49.4%	103	48.6%	25	53.2%	
N2	10	3.9%	8	3.8%	2	4.3%	
N3	18	6.9%	15	7.1%	3	6.4%	
*Pathologic T stage (ypT)*							
T0	5	1.9%	4	1.9%	1	2.1%	NA
T1	83	32.0%	61	28.8%	22	46.8%	
T1mic	3	1.2%	2	0.9%	1	2.1%	
T2	50	19.3%	41	19.3%	9	19.1%	
T3	29	11.2%	26	12.3%	3	6.4%	
T4	5	1.9%	5	2.4%	0	0.0%	
Unknown	84	32.4%	73	34.4%	11	23.4%	
*Pathologic N Stage (ypN)*							
N0	84	32.4%	64	30.2%	20	42.6%	NA
N1	54	20.8%	46	21.7%	8	17.0%	
N1mic	10	3.9%	8	3.8%	2	4.3%	
N2	21	8.1%	16	7.5%	5	10.6%	
N3	6	2.3%	5	2.4%	1	2.1%	
Unknown	84	32.4%	73	34.4%	11	23.4%	
*Breast cancer subtype*							
HR+ HER2-	140	54.1%	115	54.2%	25	53.2%	**< 0.01**
HR+ HER2+	42	16.2%	29	13.7%	13	27.7%	
HR- HER2+	10	3.9%	6	2.8%	4	8.5%	
TNBC	67	25.9%	62	29.2%	5	10.6%	
*Adjuvant endocrine therapy*							
Yes	109	42.1%	87	41.0%	22	46.8%	0.52
No	150	57.9%	125	59.0%	25	53.2%	
*Adjuvant radiation*							
Yes	113	43.6%	91	42.9%	22	46.8%	0.84
No	57	22.0%	45	21.2%	12	25.5%	
Unknown	89	34.4%	76	35.8%	13	27.7%	
Follow up^a^ (months), median (IQR)	41.0 (40.1)		40.8 (38.8)		42.3 (38.8)		0.95
Total recurrence	59	22.8%	44	20.8%	15	31.9%	0.12
Recurrence type							
Local/regional	17	6.6%	13	6.1%	4	8.5%	1.00
Distant metastasis	42	16.2%	31	14.6%	11	23.4%	
Total deceased	39	15.1%	29	13.7%	10	21.3%	0.18

Bold values indicate significant difference (*p* < 0.05)

**Table 3:** Number of patients with changed vs. unchanged biomarker profile in a 4x4 array according to pre- and post-NAC biomarker profiles (unchanged biomarker profile is in bold)

Pre-NAC	Post-NAC			
	HR+/HER2-	HR-/HER2+	HR+/HER2+	HR-/HER2-
HR+/HER2-	**115**	1	10	14
HR-/HER2+	0	**6**	3	1
HR+/HER2+	12	0	**29**	1
HR-/HER2-	5	0	0	**62**

We further reviewed the individual ER and PR statuses, particularly PR status change post-NAC in the 115 patients with a pre-NAC HR+HER2– biomarker profile. The results are presented in a 3 × 3 table, as shown in Table.S1. Of the 115 patients whose HR+HER2– status was unchanged post-NAC (*n* = 115), the majority (79, 69 %) of the PR statuses remained unchanged. For those with any ER or PR status change (*n* = 36), the most common change was conversion of PR+ to PR– status post-NAC (80 %, 29/36). Kaplan Meier event-free survival analyses comparing those converting from ER+PR+ to ER+PR– post-NAC were performed, demonstrating no significant differences between these two groups (*p* = 0.9; Fig. S1).

Whether changed biomarker profile post-NAC affected the choice of adjuvant systemic therapy is an area that is understudied. We therefore performed chart review of a subset of patients with biomarker profile change treated at the East Coast site for whom adjuvant therapy information was available (*n* = 22). Of the 12 patients with HR+HER2– disease pre-NAC, 9 had their biomarker profile changed to TNBC post-NAC, and most of these patients (6 of 9) received adjuvant capecitabine in addition to endocrine therapy. The addition of capecitabine most likely was influenced by the 2017 landmark study showing improved survival for patients who had TNBC with residual disease after NAC.^7^ All 10 of patients with HER2+ disease pre-NAC who had their biomarker profile changed to HER2– disease post-NAC continued to receive HER2-targeted therapy. It was reassuring to see that all the patients with HER2+ disease pre-NAC who had their biomarker profile changed post-NAC to HER2– continued to receive HER2-directed therapy given the results of the post hoc analyses of the Katherine Study supporting this practice.^8^

**Fig. 2:**
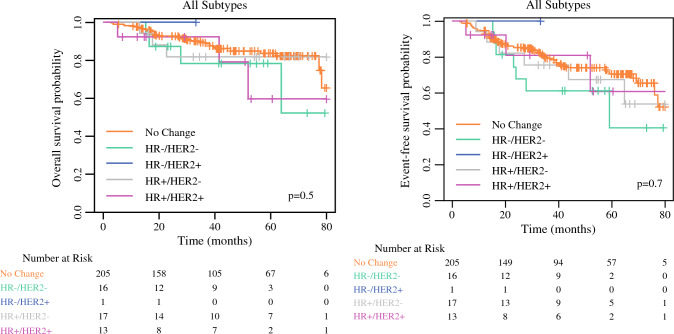
Kaplan-Meier overall survival and event-free survival analyses of cohorts stratified according to biomarker profile change status: no change vs. changed to HR+HER2-, HR-HER2+, HR+HER2+ or HR-HER2-

To evaluate whether biomarker profile change post-NAC had an impact on outcomes in our patient cohort, we performed Kaplan-Meier overall survival (OS) and event-free survival (EFS) analyses of cohorts stratified according to biomarker profile change status (no change vs change to HR+HER2–, HR–HER2+, HR+HER2+, or HR–HER2–). The results are shown in Fig. 2. Overall, OS or EFS in our patient cohort did not differ when stratified by biomarker profile status as not changed versus changed to HR+HER2–, HR–HER2+, HR+HER2+, and HR–HER2– biomarker profile post-NAC.

To further evaluate the impact of a biomarker profile change post-NAC on disease-free survival within each subtype, we performed Cox Proportional Hazards Model to compare disease-free survival hazard ratios between those with changed and those with unchanged biomarker profile in residual disease after NAC within each subtype. As shown in Table 4, we found that the conversion of two pre-NAC biomarker profiles, HR+HER2– (*n* = 14) and HR+HER2+ (*n* = 1), to TNBC was associated with worse disease-free survival, with respective hazard ratios of 2.23 (95 % CI, 0.90–5.53; *p* = 0.08, trending towards significance) and 36.7 (95 % CI, 2.2–610.8; *p* = 0.01).

**Table 4:** Cox proportional hazards model to compare disease-free survival hazard ratios with confidence interval of those with changed biomarker profile versus unchanged biomarker profile (reference) in residual disease after neoadjuvant chemotherapy

Pre-NAC	Post-NAC			
	HR+/HER2-	HR-/HER2+	HR+/HER2+	HR-/HER2-
HR+/HER2- (n=136)	**1 [Reference]**	NA	1.49 (0.35-6.43), p=0.59	2.23 (0.90-5.53), p=0.08
HR-/HER2+ (n=10)	NA	**1 [Reference]**	1.91 (0.25-14.34),p=0.53	NA
HR+/HER2+ (n=41)	0.98 (0.23-4.15), p=0.98	NA	**1 [Reference]**	36.73 (2.21-610.83), p=0.01 (n=1)
HR-/HER2- (n=65)	2.13 (0.63-7.18), 0.22	NA	NA	**1 [Reference]**

**Fig. 3:**
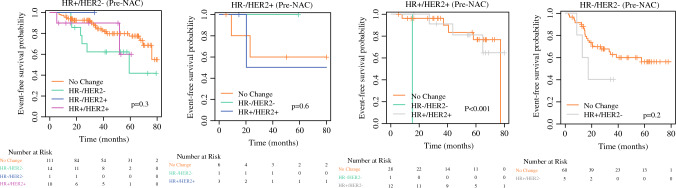
Kaplan-Meier event-free survival analyses of each of 4 pre-NAC biomarker profiles stratified according to post-NAC biomarker profile status: no change vs. changed to HR+HER2-, HR-HER2+, HR+HER2+ and HR-HER2-

We further constructed Kaplan-Meier plots of event-free survival for each of the four pre-NAC biomarker profiles stratified by changed versus unchanged biomarker profile. The results are summarized in Fig. 3. Disease-specific survival was significantly associated with biomarker profile change for those with pre-NAC HR+HER2+ disease (*p* < 0.001. The statistically significant difference was noted to be driven by one patient whose post-NAC biomarker profile changed to TNBC. Whether adjuvant treatment change would affect outcomes remains largely an unanswered question. The most significant impact may be expected for the subset of patients with HR+HER2– disease pre-NAC whose biomarker profile changed to TNBC post-NAC (*n* = 14; Fig. 3). The majority of these patients did receive adjuvant capecitabine upon further chart review. Whether the addition of capecitabine had an impact on disease-specific survival remains unclear.

## Discussion

To our knowledge, this is the largest contemporary patient cohort study examining the rate of biomarker profile change after NAC. Biomarker change was noted in 18.1 % of the patients with residual disease. Biomarker profile change was most common among the patients with HER2+ disease, at a rate of 32.7 % (17/52)**.** We found that patients with residual disease whose biomarker profile had changed from non-TNBC to TNBC experienced significantly worse outcomes. Our results demonstrating that specific biomarker profile change in residual disease has prognostic value further strengthens the rationale for re-testing of biomarker profile routinely in breast cancer patients with residual disease after NAC.

Our observed biomarker profile change rate of 18.1 % was slightly lower than the 28.8 % (36/125) reported in an earlier single-institution study by Mohan et al.,^9^ who also reported that biomarker profile change was common in HER2+ disease, with 12 patients converting from HER2+ to HER2– disease. However, the rate of biomarker conversion was unclear because the denominator or number of patients with pre-NAC HER2+ disease who did not achieve pCR was not reported in that study. In a meta-analysis that included 2847 patients from eight studies, Li et al.^10^ noted an association between biomarker profile change and clinical outcomes. Our results also showed that select biomarker profile change was associated with worse outcomes. Li et al.^10^ also reported that the conversion of HR+ to HR– status was associated with unfavorable outcomes, but it is unclear whether the worse outcomes were specific for those with biomarker conversion to TNBC. In the current study, we were able to clarify the impact of biomarker profile conversion by noting that biomarker profile change, specifically the conversion from non-TNBC to TNBC, was associated with worse event-free survival outcomes.

The strength of our study was the large patient cohort. In addition, the study cohort comprised patients treated at two large academic health centers, which enhances the generalizability of our results. One additional strength of our study was that we reported the biomarker profile change using a 4 × 4 array format, as previously described,^11^ enabling us to stratify outcome results according to subtype-specific biomarker profile change.

Despite these strengths, our study had several limitations. One limitation was our retrospective study design, with inherent selection bias and data missingness. Only about 60 % of our patients with residual disease underwent biomarker profile re-testing in this study.

Another limitation was that we did not attempt to explain the mechanism of biomarker profile change after NAC. A plausible explanation for biomarker profile change is that it results from tumor heterogeneity and tumor-editing, especially in the context of targeted therapy such as HER2-targeted therapy. In tumors with heterogeneous HER2 expression, HER2-directed therapy may have selectively eradicated HER2+ tumor cells. Our results demonstrating that biomarker profile change was most common in those with HER2+ disease, at 32.7 %, supported this hypothesis.

Other limitations included the small number of patients with biomarker profile change (*n* = 47). Due to the insufficient sample size, the significance of biomarker profile change in terms of clinical outcomes could be determined for only two of the four biomarker profiles, specifically pre-NAC HR+HER2– (*n* = 136) and pre-NAC HR+HER2+ (*n* = 42). In addition, the worse outcome related to the conversion from HR+HER2+ to TNBC, with a hazard ratio of 36.73 (95 % CI, 2.21–610.83; *p* = 0.01), should be interpreted with caution because it was derived from one patient in that subgroup of 42 patients.

Finally, the determination of pre- and post-NAC HER2 expression by immunohistochemistry may have been influenced by factors such as tissue fixation time and readers’ variability. In addition, we did not perform central pathology review. These confounding variables could not be accounted for in this study.

Our results confirmed the role of biomarker profile re-testing in residual disease, especially for those with pre-NAC HER2+ disease. The unfavorable outcomes of biomarker conversion in residual disease from non-TNBC to TNBC suggested that specific biomarker profile conversion may be prognostic and may further risk-stratify patients at increased risk of disease recurrence. For patients with pre-NAC HER2+ disease whose biomarker profile has changed to TNBC in residual disease, strong evidence indicates that they still will benefit from HER2-targeted antibody drug conjugate, as shown in a post hoc analysis by Loibl et al.^8^ Pre-clinical and clinical studies have shown that HER2-targeted antibody drug conjugate was effective for tumors with low or heterogeneous HER2 expression due to bystander tumoricidal effects.^12–14^ For patients with pre-NAC HR+HER2– disease whose biomarker profile has changed to TNBC, it is unclear whether adjuvant chemotherapy with or without immune checkpoint blockade therapy is warranted. It is even less clear whether these patients will benefit from HER2-targeted antibody drug conjugate because no data currently exist to support its use.

In summary, change in biomarker profile after NAC is especially common among patients with HER2+ breast cancer and is associated with worse outcomes when the biomarker profile is changed to TNBC profile post-NAC. Our study highlighted the variation in biomarker profile retesting in various hospital settings and the need to implement biomarker profile retesting uniformly for all patients with residual disease after NAC. Our results demonstrating that a specific biomarker profile change in residual disease may have prognostic value further strengthen the rationale for routine re-testing of biomarker profile in residual disease after neoadjuvant chemotherapy.

## Supplementary Information

Below is the link to the electronic supplementary material.

Supplementary file1 (PDF 122 KB)
