# Supplementary material for: Change in Biomarker Profile After Neoadjuvant Chemotherapy is Prognostic and Common Among Patients with HER2+ Breast Cancer
Source: Ann Surg Oncol. 2024 Aug 8;31(12):8093–101. doi: 10.1245/s10434-024-15889-3 (PMC11467097; doi:10.1245/s10434-024-15889-3)
Supplement: Supplementary file 1 — Supplementary file1 (PDF 122 KB) [file 10434_2024_15889_MOESM1_ESM.pdf]

Supplementary Table . Number of patients with pre-NAC HR+HER2- biomarker profile stratified by changed vs. unchanged PR status in a 3x3 array post-NAC (unchanged biomarker profile is shaded).

		Post-NAC		
		ER+PR+	ER+PR-	ER-PR+
Pre-NAC	ER+PR+	63	27	1
	ER+PR-	4	13	1
	ER-PR+	1	2	3

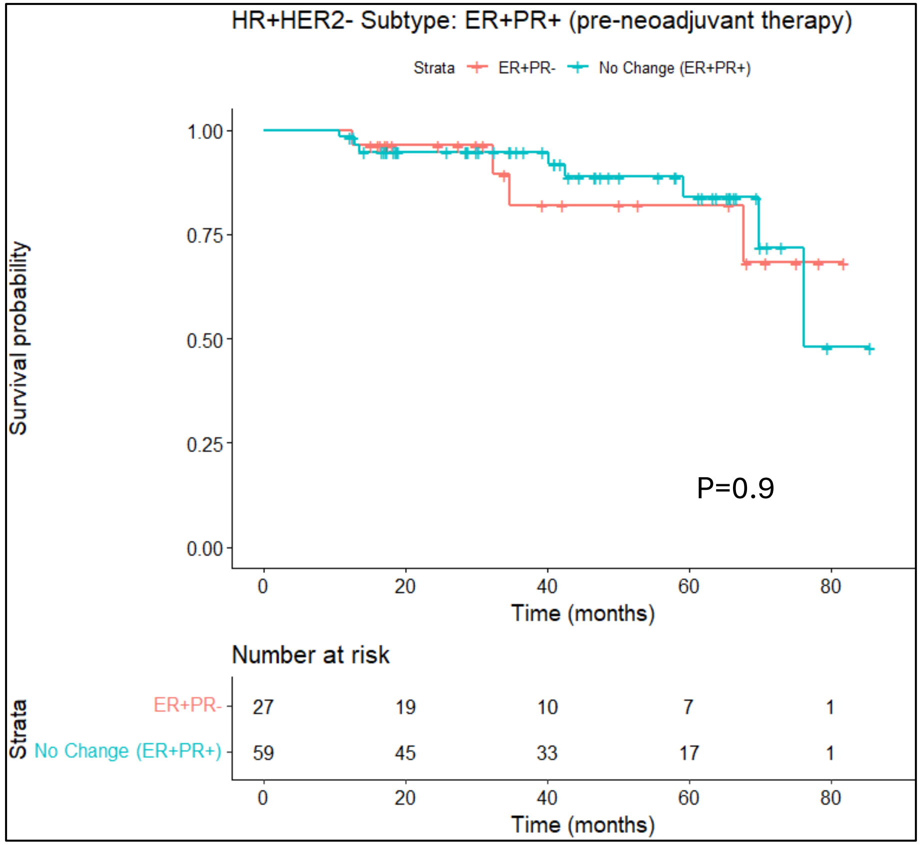

Supplementary Figure. Kaplan-Meier event-free survival analyses of patients with pre-NAC HR+HER2- biomarker profile stratified by changed vs. unchanged PR status
